# Healthy lifestyles and wellbeing reduce neuroinflammation and prevent neurodegenerative and psychiatric disorders

**DOI:** 10.3389/fnins.2023.1092537

**Published:** 2023-02-15

**Authors:** Elodie Kip, Louise C. Parr-Brownlie

**Affiliations:** Department of Anatomy, School of Biomedical Sciences, Brain Health Research Centre, Brain Research New Zealand, University of Otago, Dunedin, New Zealand

**Keywords:** neuroinflammation, healthy lifestyle, wellbeing, neurodegeneration, psychiatric, reduce risk

## Abstract

Since the mid-20th century, Western societies have considered productivity and economic outcomes are more important than focusing on people’s health and wellbeing. This focus has created lifestyles with high stress levels, associated with overconsumption of unhealthy foods and little exercise, which negatively affect people’s lives, and subsequently lead to the development of pathologies, including neurodegenerative and psychiatric disorders. Prioritizing a healthy lifestyle to maintain wellbeing may slow the onset or reduce the severity of pathologies. It is a win-win for everyone; for societies and for individuals. A balanced lifestyle is increasingly being adopted globally, with many doctors encouraging meditation and prescribing non-pharmaceutical interventions to treat depression. In psychiatric and neurodegenerative disorders, the inflammatory response system of the brain (neuroinflammation) is activated. Many risks factors are now known to be linked to neuroinflammation such as stress, pollution, and a high saturated and trans fat diet. On the other hand, many studies have linked healthy habits and anti-inflammatory products with lower levels of neuroinflammation and a reduced risk of neurodegenerative and psychiatric disorders. Sharing risk and protective factors is critical so that individuals can make informed choices that promote positive aging throughout their lifespan. Most strategies to manage neurodegenerative diseases are palliative because neurodegeneration has been progressing silently for decades before symptoms appear. Here, we focus on preventing neurodegenerative diseases by adopting an integrated “healthy” lifestyle approach. This review summarizes the role of neuroinflammation on risk and protective factors of neurodegenerative and psychiatric disorders.

## 1. Introduction

Globally, the human lifespan is longer than it has been historically. Given that the risk of neurological disease increases dramatically with age, the incidence of these conditions has seen a similar increase. As a result, neurological disorders affect millions of people worldwide and collectively are the second leading cause of death (9 million) and a primary cause of disability resulting in a substantial global health and socioeconomic burden (GBD 2016 Neurology Collaborators, 2019). The Global Burden of Disease Study reported that the prevalence of dementia, stroke, and parkinsonism increased two to three times between 1990 to 2016 due to the aging population (GBD 2016 Neurology Collaborators, 2019). The Global Burden of Disease Study also showed that the two most common neurodegenerative diseases in 2016 were dementia and Parkinson’s disease (PD) (GBD 2016 Neurology Collaborators, 2019), affecting 43.8 and 6.1 million people worldwide, respectively (GBD 2016 Dementia Collaborators, 2019) (GBD 2016 Parkinson’s disease Collaborators, 2018). These statistics highlight the urgent need to elucidate the causes of neurological and neurodegenerative diseases, and how they can be prevented and treated more effectively.

For neurological disorders, brain, spinal cord and peripheral nerve functions are altered, plus their associated movement, sensory, or memory functions. Neurodegenerative diseases are a subset of neurological diseases in which neurons in the brain, spinal cord, or peripheral nervous system are progressively damaged or die, thereby altering function. Psychiatric conditions are brain disorders in which regions that control emotions, mood, identity, consciousness and thinking are altered (Baker et al., 2002; David and Nicholson, 2015), causing depression, schizophrenia, bipolar disorder, anxiety disorders, neurodevelopmental disorders (autism, attention deficit hyperactivity disorder), addictive disorders, and stress-related disorders. While neurological, neurodegenerative, and psychiatric disorders are categorized separately, an individual may present with symptoms across categories, e.g., neurodegenerative disease symptoms may present with psychiatric symptoms (David and Nicholson, 2015). Moreover, sometimes psychiatric disorders might occur after being initiated by a neurological disorder, or the other way around, showing the interconnection between these conditions. The etiologies of neurological, neurodegenerative, and psychiatric diseases are complex, poorly understood, but often include genetic and environmental factors. Including all neurological disorders would be too extensive for this review, therefore, we focus on neurodegenerative disorders and some psychiatric conditions.

Neurodegenerative disorders are irreversible due to the poor capacity for neurons to undergo mitosis to regenerate, and can be age-related. The mechanisms of neurodegenerative diseases involve multiple common pathologies like accumulation of misfolded proteins, impaired cell organelle function, and neuroinflammation (Katsnelson et al., 2016). In older people, innate and adaptive immune responses are impaired, leading to chronic low-grade inflammation that is hypothesized to accelerate biological aging; a process dubbed “inflammaging” (Magrone et al., 2020). Neuroinflammation in the central nervous system (CNS) occurs in neurodegenerative diseases, and also underlies psychiatric diseases such as depression and schizophrenia (Nettis and Pariante, 2020; Murphy et al., 2021; Troubat et al., 2021). Neuroinflammation is linked to perturbations of the peripheral immune system. An important strategy to avoid neuronal damage and subsequent development of neurodegenerative and psychiatric disorders is to moderate peripheral and neuroinflammation, i.e., decrease risk factors and/or increase protective factors, for example, by consuming a healthy diet and exercising regularly throughout the lifespan. Once symptoms have presented, treatments involve relieving physical and psychiatric symptoms, and it is often too late to reverse the underlying causes of the disease. This highlights the critical need for future research to focus on early pathophysiology mechanisms to prevent or minimize irreversible damage.

The objective of this review is to examine new approaches to manage neurodegenerative and psychiatric disorders that work by reducing or preventing neuroinflammation. First, neurodegenerative diseases and their multifactorial etiology are discussed. Second, we describe the role of neuroinflammation in neurodegenerative and psychiatric disorders and discuss common mechanisms. Third, we discuss risk and protective factors involved in neuroinflammation. Overall, we highlight the importance of preventing neuroinflammation-related pathologies that underlie neurodegenerative and psychiatric disorders, which are increasingly prevalent world-wide.

## 2. Neurodegenerative diseases have multifactorial etiology

**FIGURE 1:**
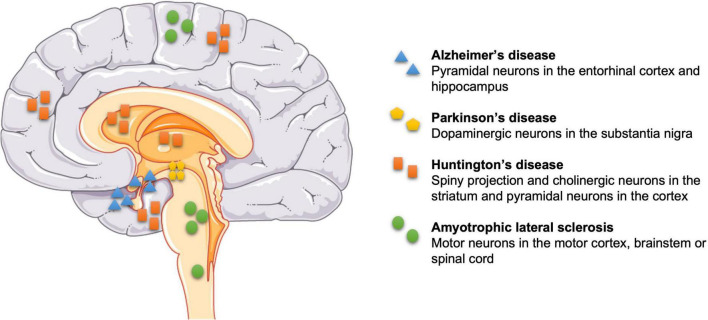
Selective sites of neuronal death in some neurodegenerative diseases. Pyramidal neurons in the entorhinal cortex layer II and hippocampus layer CA1 in Alzheimer’s disease (AD), dopaminergic neurons in the substantia nigra pars compacta in Parkinson’s disease (PD), GABAergic spiny projection and cholinergic neurons in the striatum, and pyramidal neurons in the cortex in Huntington’s disease (HD), and motor neurons in the motor cortex, brainstem or spinal cord in amyotrophic lateral sclerosis (ALS).

Neurodegeneration is the term to describe early and progressive loss of specific neurons, and altered associated functions, within the CNS and periphery (Subramaniam, 2019). Neuronal death can cause Alzheimer’s disease (AD), PD, Huntington’s disease (HD), and amyotrophic lateral sclerosis (ALS), which pose substantial health, wellbeing and treatment challenges because once the neurodegeneration has started, progression can only be slowed but not suppressed or reversed (Fu et al., 2018). Figure 1 shows examples of neuronal death linked to diseases: degeneration of neurons in the entorhinal cortex layer II (ECII) and hippocampus layer CA1, plus also frontal, parietal and temporal lobes of the cortex (Mukhin et al., 2017) impair memory in AD (Van Hoesen et al., 1991); degeneration of dopaminergic neurons in the substantia nigra pars compacta (SNpc) impairs (slow) movements and causes rigidity and tremors in PD (Hughes et al., 1992); loss of GABAergic spiny projection neurons (also known as medium spiny neurons) and cholinergic neurons in the striatum, and glutamatergic pyramidal (principal) neurons across the whole cerebral cortex are associated with involuntary movements in HD (Rikani et al., 2014; Blumenstock and Dudanova, 2020); and loss of motor neurons in the motor cortex, brainstem, or spinal cord that control skeletal muscles underlie ALS.

A hallmark of neurodegenerative diseases is the formation of misfolded protein aggregates, which seem to trigger neuronal death. For example, accumulation of amyloid beta peptide and/or tau is associated with AD (Van Hoesen et al., 1991), α-synuclein (α-syn) with PD, Huntingtin (HTT) protein aggregates occur in HD and superoxide dismutase 1 protein aggregates (SOD1) with ALS (Sweeney et al., 2017). Moreover, several common pathophysiological mechanisms are found early in the disease course and also lead to cell death including mitochondrial dysfunction, lysosomal depletion, impaired RNA homeostasis, altered glial function, and increased neuroinflammation.

Aging, genetics, the environment, and the interaction of these factors play a role in disease onset and progression. A detailed description of the vast number of genetic factors and their impact on aging is beyond the scope of this review, but there are some common facts across neurological diseases. With aging, neurons in all regions of the nervous system are affected, such as diminished pyramidal neuronal populations in CA1 layer in the hippocampus, principal neurons of ECII, spiny projection neurons in the striatum and dopaminergic neurons in the SNpc (Mattson and Magnus, 2006; Hou et al., 2019). These changes underlie the age-related decline of sensory, motor, and cognitive functions.

Genetic factors are also central to neurodegeneration etiology. Examples of hereditary genes and their corresponding disease are: *HTT* gene in HD, amyloid precursor protein (*APP*), presenilin (*PSEN1*, *PSEN2*), apolipoprotein E (*APOE*) genes in AD, chromosome 9 open reading frame 72 (*C9org72*), *SOD1*, and TAR DNA binding protein (*TARDBP*) genes in ALS (Pihlstrom et al., 2017). Genes involved with PD are parkin 2 (*PARK2* or *PRKN*), gene encoding α-syn (*SNCA* or *PARK1*), leucin-rich repeat kinase 2 (*LRRK2* or *PARK8*), glucocerebrosidase (*GBA* or *PARK18*), PTEN induced putative kinase 1 (*PINK1* or *PARK6*), protein deglycase DJ-1 (or *PARK7*), and PARK-vacuolar protein sorting ortholog 35 (*VSP35* or *PARK17*) (Klein and Westenberger, 2012).

Environmental and lifestyle factors that are known to promote neurodegeneration are exposure to pesticides, heavy metals, air pollution, a diet high in saturated fat, chronic stress, gut inflammation, and virus infections (Brown et al., 2005; Kip and Parr-Brownlie, 2022). By better understanding how the interplay of age, genetics, and exposure to environmental and lifestyle factors alters protein formation, triggers neurodegeneration, and promotes neuroinflammation, alongside the development of tools to diagnose diseases at prodromal stages, we can develop novel approaches to prevent, stop, or slow disease progression.

## 3. Neuroinflammation plays a role in neurodegenerative and psychiatric diseases

In the past, neurological disorders have focused on neuronal dysfunction and degeneration, but this has been replaced in recent years with a more nuanced perspective, including involvement of glia, immune cells, and inflammatory processes in the pathology.

**FIGURE 2:**
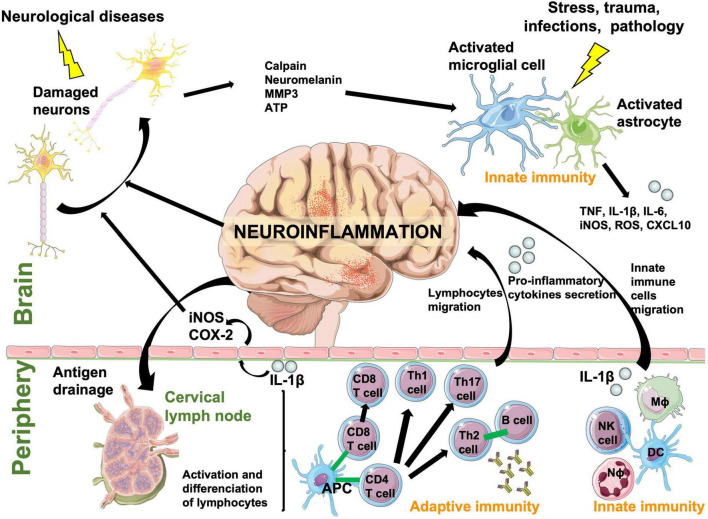
A schematic diagram showing the bidirectional communication between central and systemic immune system responses that cause neuroinflammation. Resident immune cells in the brain, microglia and astrocytes, act as scavengers to rapidly react to a pathological event. Immune cells in the periphery communicate with microglia and astrocytes through cytokines and chemokines. Foreign/pathological substances or antigens are drained from the CNS to peripheral lymph nodes where they are processed and presented to adaptive cells by antigen presenting cells. Peripheral innate and adaptive cells react to these signals and antigen presentation and cross the blood brain barrier to help eliminate the pathological event. They are also responsible of neuronal damage by inducing neuroinflammation. APC, antigen-presenting cell; ATP, adenosine triphosphate; CD4 T cell, lymphocyte T helper expressing CD4 (cluster of differentiation 4) glycoprotein; CD8 T cell, cytotoxic T cell expressing CD8; COX-2, cyclooxygenase-2; CXCL10, C-X-C motif chemokine ligand 10; DC, dendritic cell; IL, interleukin; iNOS, inducible nitric oxide synthase; Mϕ, macrophage; MMP3, matrix metalloproteinase-3; Nϕ, neutrophil; NK, natural killer; Th cell, lymphocyte T helper; TNF, tumor necrosis factor.

The immune response is the natural defense system of the body. When too robust, it can damage surrounding tissues and cause autoimmune pathologies, especially within the brain where neuronal regeneration is poor. Leukocytes are largely absent from the healthy CNS parenchyma and are mostly located in the meninges and choroid plexus (Korin et al., 2017), unlike microglia and astrocytes, which are resident scavenger immune cells of the brain. The innate immune system rapidly and non-specifically eliminates pathogens and foreign molecules or organisms using inflammation. In the periphery, the innate immune system includes neutrophils, monocytes/macrophages, dendritic cells and natural killer cells, while in the brain this function is fulfilled and initiated only by resident microglia and astrocytes (Crotti and Ransohoff, 2016). When microglia and astrocytes are activated by stress, trauma, pathology, or infection, they secrete reactive oxygen species (ROS), pro-inflammatory cytokines, and chemokines facilitating recruitment of myeloid and lymphoid cells into the brain (Ransohoff and Brown, 2012). Microglial and astrocyte activation also induces recruitment of adaptive immune cells specific to the pathological agent, such as B and T lymphocytes, because adaptive immunity cannot be initiated directly in the brain. The CNS can also respond to peripheral inflammatory stimuli and initiate a local inflammatory state in the brain (Figure 2). Cytokines such as interleukin-1 (IL-1) circulating in the blood or lining blood vessels can signal the inflammatory state to neurons *via* substances such as nitric oxide (NO), which is synthetized from the inducible isoform of the nitric oxide synthase (iNOS) and cyclooxygenase (COX-2) in endothelial cells lining blood vessels. Therefore, inflammatory signals can be transmitted from the blood to neurons without IL-1β crossing the blood brain barrier (BBB) (Licinio and Wong, 1997). Then, bidirectional communication between the brain and systemic immune system eliminates pathogens, foreign molecules, and organisms (Ransohoff and Brown, 2012).

**FIGURE 3:**
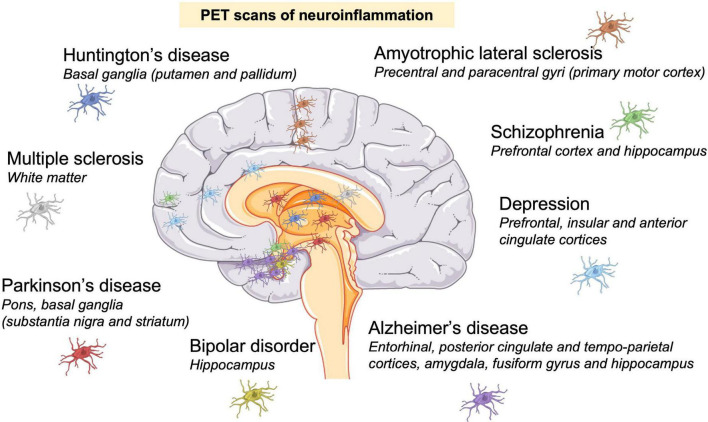
Neuroinflammation is a hallmark of neurodegenerative and psychiatric disorders. Neuroinflammation was identified by positron emission tomography (PET) scans in patients. Presence of activated microglia (colored cells) in: Huntington’s disease (Lois et al., 2018); Parkinson’s disease (Gerhard et al., 2006); multiple sclerosis (Datta et al., 2017); amyotrophic lateral sclerosis (Alshikho et al., 2018); Alzheimer’s disease (Cagnin et al., 2007; Valotassiou et al., 2018); depression (Setiawan et al., 2015), schizophrenia (Doorduin et al., 2009; Marques et al., 2017); and bipolar disorder (Haarman et al., 2014).

When optimal communication between the brain and systemic immune system fails to occur at the appropriate time, inflammatory mediators accumulate in the CNS in a process known as neuroinflammation and can trigger brain damage. Damaged neurons release cytosolic factors that will cause further microglial and astrocyte activation, which will exacerbate neuroinflammation and neuronal damage (Figure 2). This becomes a vicious cycle, and when this occurs chronically contributes to the development and maintenance of chronic pain, neuropsychiatric disorders like schizophrenia and depression, and neurodegenerative diseases (Wee Yong, 2010; Fusco et al., 2017; Figure 3). Neuroinflammation occurs and is a key element in neurodegenerative diseases such as PD, AD, HD, ALS, and multiple sclerosis (MS), and has been well described by Suescun et al. (2019). Stopping neuroinflammation early, and before it amplifies and becomes chronic, will be key to preventing many neurodegenerative diseases (Kip and Parr-Brownlie, 2022). Furthermore, peripheral inflammation needs to be controlled. In response to peripheral inflammation, neuroinflammation is activated and can trigger psychiatric disorders like depression and anxiety in otherwise healthy people. These diseases were largely thought to be based on dysregulation of neurotransmitter systems, but neuroinflammation is now believed to be a key element. Depression is one of the most common and costly of all neuropsychiatric disorders, and is also associated with an increased risk for diseases that have an immunological basis such as asthma, rheumatoid arthritis, chronic pain, systemic infections, autoimmune diseases, cancer, and neurodegenerative diseases (Dantzer et al., 2008; Harkness et al., 2019). Animal studies, *postmortem* analysis and epidemiological studies have documented that CNS immune system dysregulation is linked to depression, anxiety, cognitive dysfunction, and sleep impairment. Inflammation induces several symptoms of depression in individuals, such as fatigue, anorexia, pain and sleep disorders, depressed mood, anxiety, and irritability (Dantzer et al., 2008; Miller et al., 2009; Slavich and Irwin, 2014). Moreover, pro-inflammatory cytokines such as IL-1β and IL-6 are increased in the serum of patients with depression and interferon gamma (IFN-γ) is increased in bipolar disorder. Drugs that target neuroinflammation, such as COX-2 inhibitors, are also now considered treatments for psychiatric diseases (Lucas et al., 2006).

Imaging microglial activation *in vivo* using position emission tomography (PET) has been a valuable tool to determine the location of neuroinflammation in neurological and psychiatric disorders in patients. The main imaging target for PET studies is the 18 kDa translocator protein (TSPO), which is expressed by activated microglia, and individual TSPO PET tracers have limitations and advantages (Sridharan et al., 2017; Werry et al., 2019). Figure 3 shows PET-identified neuroinflammation sites in some neurodegenerative and psychiatric disorders. Neuroinflammation sites usually correspond to the site of neuronal death, confirming neuroinflammatory mechanisms in these pathologies.

Aging is also associated with increased neuroinflammatory processes. Indeed, increased basal levels of pro-inflammatory cytokines such as IL-6, inflammasome activation and reduced levels of anti-inflammatory cytokines such as IL-10 are observed with age, a phenomenon called “inflammaging” (Ye and Johnson, 1999; Cribbs et al., 2012; Magrone et al., 2020). Therefore, it is logical that neuroinflammation is linked to age-related neurodegenerative diseases such as AD and PD because the majority of cases occur in people over 50 years of age. Many scientists believe that neurodegenerative diseases are only linked to abnormal aging processes, however, neuroinflammation during early stages of life is also linked to later life neurodegenerative diseases (Eikelenboom et al., 2010; Stokholm et al., 2017). Therefore, preventing or minimizing neuroinflammation throughout all stages of life may proactively and cumulatively reduce the risk of developing neurodegenerative and psychiatric disorders, improve general health, and reduce the individual and society cost of managing these diseases (Kip and Parr-Brownlie, 2022).

## 4. Risk and protective factors for neuroinflammation in neurodegenerative and psychiatric diseases

**FIGURE 4:**
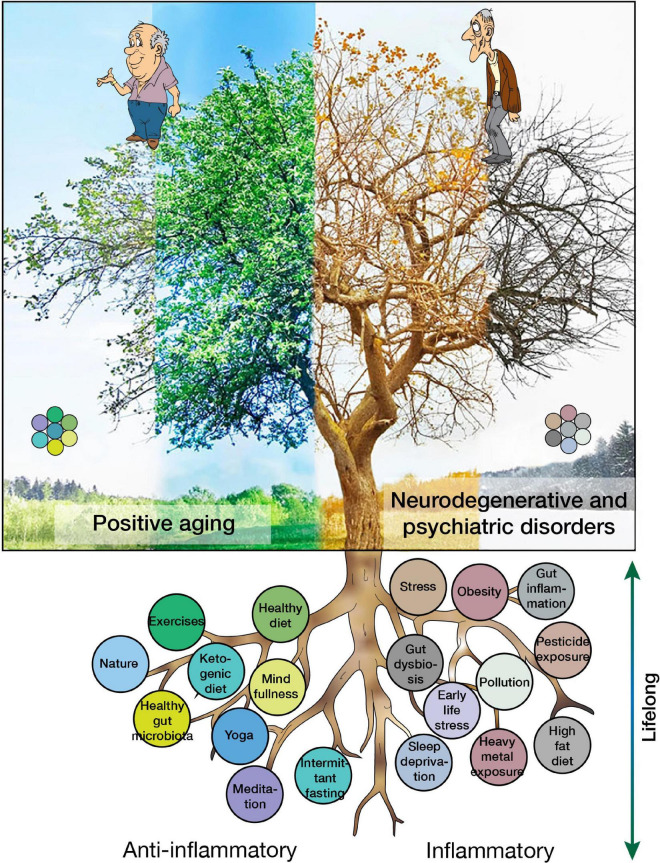
Risk and protective factors for neuroinflammation and their contributions to positive aging or the development of neurodegenerative and psychiatric disorders.

There are many factors that can exacerbate or prevent neuroinflammation, thereby increasing or decreasing the risk for PD as described in our recent review (Kip and Parr-Brownlie, 2022), but also neurodegenerative and psychiatric diseases in general (Figure 4). The most common modifiable risk and protective factors are detailed below.

### 4.1. Physical activity

A lot of people spend much of their time at work and home on computers, engaging *via* smart phones and other devices, and watching TV. A sedentary lifestyle and physical inactivity are identified by the World Health Organization (WHO) as the fourth leading risk factor for global mortality. A sedentary lifestyle increases the risk of cardiovascular disease, diabetes and cancer and their associated risk factors such as high blood pressure, raised plasma glucose levels and being overweight (Dietz, 1996; Hu, 2003; Lavie et al., 2019). In contrast, extremely intense exercise can cause overtraining syndrome with a variety of symptoms, both physical and psychological. It also suppresses the immune system, and can cause adverse cardiovascular effects and exercise addiction, which is an unhealthy compulsion to exercise (Nieman, 2000; World Health Organization, 2010; Freimuth et al., 2011; O’Keefe et al., 2012).

Moderate exercise is defined as exercises that get the heart rate 50–60% higher than its resting rate. Moderate exercise reduces sedentary-induced side effects (Jakicic and Davis, 2011; Lavie et al., 2015; Stout et al., 2017). Immediately after exercise, many positive outcomes are observed such as lower blood pressure, less stress and anxiety, better sleep and improved mood. This shows that moderate exercise influences most functions in the body, including the CNS, where it improves brain function on acute and chronic timescales, induces release of neurotransmitters, neurotrophic factors, and stimulates neurogenesis (Deslandes et al., 2009) and neuroplasticity (Cassilhas et al., 2016).

Current evidence supports the disease-altering potential of exercise through modification of neuroimmune responses in major depressive disorders, schizophrenia, AD, and PD, which is well described by Spielman et al. (2016). Physical activity inhibits inflammation by suppressing microglial activation and inducing an anti-inflammatory state. In more detail, physical activity in animal models and humans increases anti-inflammatory factors [anti-inflammatory cytokines, cluster of differentiation 200 and its receptor (CD200-CD200R), triggering receptor expression on myeloid cells 2 (TREM2), heat-shock proteins (HSP), metabolic factors, brain-derived neurotrophic factors, anti-oxidants, stimulating the glymphatic system] and decreases pro-inflammatory factors [pro-inflammatory cytokines IL-1β and TNF-α, and chemokines chemokine (C-C motif) ligand 2 (CCL2) and C-X-C motif chemokine ligand 10 (CXCL10), and toll-like receptor signaling pathway] (Mee-Inta et al., 2019). Physical activity in AD animal models decreases cellular and cognitive impairment by modulating neuroinflammation (Kelly, 2018; Liu et al., 2020). Outcomes were also observed in animal models of PD by decreasing pro-inflammatory factors and microglial activation, decreasing dopaminergic cell loss; thereby reducing akinesia, and improving motor coordination (Tillerson et al., 2003; Tajiri et al., 2010; Sung et al., 2012; Real et al., 2017). In HD animal models, exercise reduced striatal neuron loss, improved motor coordination and delayed cognitive decline (Pang et al., 2006; Harrison et al., 2013). In MS animal models, exercise decreased pro-inflammatory cytokines and increased anti-inflammatory cytokines, and also attenuated the clinical score (Bernardes et al., 2013; Zaychik et al., 2021).

Exercise is also good for overall mental health by improving memory, cognition, sleep, and mood, and decreasing psychiatric symptoms like depression, stress, anxiety, and mood disorders. Here, its affects are thought to be due to the production of neurotrophic factors, neurotransmitters, hormones, and growth of new CNS blood vessels, and by reducing neuroinflammation (Mahalakshmi et al., 2020). A study with college students showed that moderate-intensity exercise reduced symptoms of stress and depression while reducing levels of TNF-α (Paolucci et al., 2018). In this regard, the COVID-19 pandemic induced isolation and quarantine requirements contributed to the increased prevalence of psychiatric disorders such as anxiety and depression, and exercise is a valuable tool to combat it (Hu et al., 2020).

Finally, physical activity can also improve the composition of the gut microbiome, decreasing peripheral and central inflammation, like microglial activation in the CNS (Abraham et al., 2019; Gubert et al., 2020). Evidence shows that there is a reciprocal link between a reduction of peripheral inflammation and neuroinflammation. This explains the combined impact of a healthy diet and moderate exercise to prevent peripheral inflammation, neuroinflammation, neurodegenerative, and psychiatric disorders.

### 4.2. The gut-brain axis

Although there is anatomical separation, recent evidence indicates that neuroinflammation in the brain might originate in the intestine through communication between the enteric and CNS, which is called the gut-brain axis. Dietary intake determines the composition of the gut microbiota. Therefore, the precise content of the food eaten may affect brain health more directly than previously thought.

Gut microbiota dysbiosis is linked to diseases within the entire body (Petersen and Round, 2014; Novakovic et al., 2020; Sencio et al., 2021). The levels and balance of dietary elements such as fat, carbohydrate, gluten, alcohol, vitamins, and food allergens have a role in determining gut microbiota composition and whether inflammation is triggered in the gut. In the CNS, gut dysbiosis is associated with impaired mood, anxiety, depression, pain and impaired cognition and directly linked to neuroinflammation (Cryan and Dinan, 2012). Furthermore, chronic intestinal inflammation has been linked to neuroinflammation, and development of neurodegenerative diseases (Houser and Tansey, 2017) and bipolar disorder (Muneer, 2016).

Several dietary components, when eaten in excess, have been linked to gut inflammation and neuroinflammation. Western diets that are high in red meat, saturated and *trans* fats, refined sugars and carbohydrate intake are linked to gut inflammation and a change in microbiota composition (Agus et al., 2016). In contrast, other nutritional components (unsaturated, polyunsaturated, and monounsaturated fat) and dietary habits such as ketogenic and Mediterranean diets, and intermittent fasting, are neuroprotective and anti-inflammatory. Adherence to some anti-inflammatory promoting diets can be challenging over long periods. Therefore, instead of suppressing components from the diet, balancing anti-inflammatory foods and habits may be critical to reduce neuroinflammation.

In the past, fats were considered bad for health, but now their health effects are nuanced depending on the type of fat. High consumption of some fats increases the risk of heart disease and diabetes (Siri-Tarino et al., 2010; Schlesinger et al., 2019), whereas other fats are needed in our diet and have a lower cardiovascular risk profile. Dietary fats differ in their chemical structure, and therefore, the extent that they trigger inflammation and neuroinflammation. Saturated and *trans* fats have the maximum number of hydrogen atoms bound to carbon atoms and are solid at room temperature. They are considered the least healthy fat, and are found in many foods, such as red meat, butter, palm and coconut oils, milk, cheese, ice cream, French fries and crackers (Kris-Etherton et al., 2012; de Souza et al., 2015). Unsaturated fats have fewer hydrogen atoms bound to carbon atoms, mono-unsaturated fats have one unsaturated bond, whereas polyunsaturated fats have many. Mono and polyunsaturated fats are healthy and found in vegetables, nuts, fish (mono); avocado, olive and canola oil, almonds (unsaturated); walnut, sunflower oil, salmon, tuna (poly). There are two types of polyunsaturated fats - omega-3 and omega-6 fatty acids. Omega-3 fatty acids are found in oily fish, nuts, flaxseeds and leafy vegetables, and are considered the healthiest fat because they may reduce inflammation (Murphy et al., 2021; Saini et al., 2021).

A common impact of excessive consumption of unhealthy fats and foods is an increase in neuroinflammation. A high fat diet (HFD) that contains a lot of saturated and *trans* fats, promotes pro-inflammatory changes in the small intestine, and activates the toll-like receptor 4 (TLR4), iNOS, COX-2, and nuclear factor kappa-light-chain-enhancer of activated B cells (NF-κB) (Kim et al., 2012). Subsequently, a HFD induces oxidative stress, neuroinflammation, tau hyperphosphorylation and neuron degeneration (Anstey et al., 2011), plus microglial activation (Kim et al., 2019). In animal models, a HFD induces cognitive decline similar to the symptoms of AD, and amyloid accumulation on artery walls in the brain (Lin et al., 2016).

Another nutrient that can trigger inflammation is gluten. People, even non-celiac, can be sensitive to gluten and gluten intolerance and sensitivity are often unrecognized and under-diagnosed. Daulatzai (2015) reported that non-celiac gluten sensitivity can trigger gut dysbiosis, dysregulating the gut-brain axis by increasing intestinal permeability, entry of bacteria, bacterial toxins, and toxic digestive metabolites into the bloodstream, increasing neuroinflammation and BBB permeability, and subsequently increase the risk for an individual to develop dementia. PD, AD, depression, anxiety, and schizophrenia are also believed to be linked to gluten intake, especially in celiac patients (Levinta et al., 2018; Mohan et al., 2020). However, data are limited and further research is needed (Philip and White, 2022).

Epidemiological and animal studies have revealed the broad underlying role that neuroinflammation may have in neurodegenerative disorders, regardless of the initiating trigger or events. For example, metabolic disorders, such as obesity, activate neuroinflammation and are correlated to neurodegenerative disorders (Maric et al., 2014; Procaccini et al., 2016; Mazon et al., 2017), and bariatric surgery treatment reduces low grade inflammation (Rao, 2012; Stolberg et al., 2018). When placed in the context that there is a worldwide epidemic of obesity, it remains critical to eat healthy foods that reduce inflammation and avoid foods that negatively change the composition of gut microbiota and trigger inflammation, such as a HFD.

Anti-inflammatory dietary components are found in many foods and plants, and they are well described in a recent review (Rekatsina et al., 2020). Naturally occurring common anti-inflammatories are found in pomegranates, medicinal plants, vitamin D, vitamin C, a Mediterranean diet and extra-virgin olive oil, flavonoids found in fruits, curcumin, resveratrol, aged garlic extract, walnuts, marine carotenoid astaxanthin found in seafood, omega-3 fatty acids, caffeine, and manuka honey (Almasaudi et al., 2017). Ingestion of these foods reduce chronic inflammation and neuroinflammation (Rekatsina et al., 2020; Di Majo et al., 2022; Itsiopoulos et al., 2022). These (neuro)inflammation reducing foods could be part of the prevention and treatment of neurodegenerative and psychiatric disorders, however, clinical studies are needed. A specific dietary example is salvianolic acid B (sal B), which is extracted from salvia miltiorrhiza bunge, a popular Chinese herb. Sal B abolishes neuroinflammatory responses in the hippocampus in a rat model of chronic mild stress-induced depression by inhibiting NOD-, LRR-, and pyrin domain-containing protein 3 (NLRP3) inflammasome activation (Huang et al., 2019). Sal B also reduces MS severity by impairing Th1 and Th17 responses, limiting astrogliosis and infiltration of inflammatory cells into the CNS in a MS animal model (Dong et al., 2016). Sal B has anti-inflammatory effects in a model of traumatic brain injury–brain edema and motor deficits were reduced by inhibiting neutrophil infiltration, microglia activation, and pro-inflammatory cytokine production (Chen et al., 2011).

Recently, intermittent fasting (IF) and ketogenic diets (KD) have been shown to positively impact gut microbiota and increase people’s health and wellbeing. IF is a practice associated with weight loss and calorie restriction where individuals refrain from eating for extended periods of the day, e.g., 16 h per day (16:8 ratio). In addition to facilitating weight loss, IF has been shown to increase lifespan, reduce free radicals, attenuate age-related diseases and reduce cognitive and motor function decline (Mattson, 2005; Mattson and Wan, 2005; Masoro, 2006; Gudden et al., 2021). IF stimulates adaptive immune responses in rats, which suppress LPS-induced neuroinflammation in young and old animals. These results support the idea that IF could reduce the risk of neuroinflammation at any point in the life span, and cumulatively could significantly reduce the risk of neurodegenerative diseases linked to inflammatory responses (Vasconcelos et al., 2015). IF also decreases plasma inflammatory factors such as cortisol, IL-6 and TNF-α in a mouse model of stress, which was hypothesized to ameliorate cognitive function (Shojaie et al., 2017). In a MS animal model, IF reduced inflammation, increased bacteria richness in the gut and ameliorated the clinical course of the disease, which has also been shown in MS patients (Cignarella et al., 2018). Similarly, in a mouse model of PD, IF attenuated dopaminergic neuron loss by upregulating neurotrophic factors and decreasing neuroinflammation (Ojha et al., 2023).

A ketogenic diet (KD) is a high fat, low carbohydrate diet with adequate levels of protein that shifts the body from glucose to fat metabolism. As a consequence, the liver converts fats into ketones, which can serve as a major energy source for the brain. A KD for 12 days and 8 weeks improved the quality of life and daily function in patients with AD (Phillips et al., 2021) and PD (Phillips et al., 2018), respectively, and helped patients living with severe epilepsy (Fan et al., 2019). Emerging evidence shows that a KD induces systemic and neuroprotective anti-inflammatory effects. In rodent models of neurodegenerative and neuro-inflammatory disorders, a KD can reduce expression of pro-inflammatory cytokines and microglial activation (Ruskin et al., 2009; Yang and Cheng, 2010; Youm et al., 2015) probably by activating the peroxisome proliferator-activated receptor α which inhibits NF-κB, leading to the downregulation of COX2 and iNOS (Cullingford, 2004), and/or by directly inhibiting the NLRP3 inflammasome (Youm et al., 2015). A KD is thought to improve psychiatric diseases such as mood disorders (Brietzke et al., 2018) and depression (Włodarczyk and Cubała, 2019) through anti-inflammatory affects. However, large scale studies and clinical trials are required to fully assess the impact of KD on the treatment or management of neurodegenerative and psychiatric disorders (Jensen et al., 2020), and if there are other mechanisms of introducing ketonemia.

The gut-brain axis likely plays a key role in the development and progression of brain disorders. Other dietary compounds that maintain healthy microbiota and inflammation levels in the gut will also be important such as caffeine, probiotics and prebiotics (Frank et al., 2019), and could also be used as prevention and treatment strategies. Nevertheless, food quality and quantity can be used to optimize health and wellbeing throughout the lifespan.

### 4.3. Alcohol consumption

Chronic alcohol consumption alters the gut microbiome, causes mucosal damage due to increased permeability to endotoxins, and causes systemic inflammation by activating monocytes and macrophages to secrete TNF-α and other pro-inflammatory cytokines (Bode and Bode, 2005; Amin et al., 2009). Systemic inflammation is a precursor of neuroinflammation, therefore, chronic alcohol consumption is also linked to the development of neurodegeneration (Qin et al., 2008; Qin and Crews, 2012; Kamal et al., 2020).

Recent epidemiological studies show that compared to people who abstain from alcohol consumption, light-moderate alcohol consumption in any form (i.e., wine, beer, or spirits) can be beneficial for health (Kamal et al., 2020). Moderate drinking is defined as a maximum of two standard drinks daily for women, and three for men. Several studies report that low alcohol consumption can promote anti-inflammatory and cytoprotective processes by reducing C-reactive protein (CRP) levels, and plasma markers of inflammation and pro-inflammatory cytokines such as IL-6 (Albert et al., 2003; Vasanthi et al., 2012; Justice et al., 2018). On top of that, case-control and cohort studies show moderate alcohol consumption can lower the risk of dementia (Ruitenberg et al., 2002; Mukamal et al., 2003; Pilleron et al., 2015; Sabia et al., 2018; Radford et al., 2019). Polyphenolic components of red wine, such as resveratrol, reduce toxicity in an animal model of PD through their antioxidant and anti-inflammatory properties. This reinforces the importance of moderate alcohol consumption as part of a balanced lifestyle for health.

### 4.4. Mental health and wellbeing

Stress is a state of the body and mind that occurs when an alerting, demanding, or threatening event occurs. The stress response is adaptive and can be behavioral, psychological, or physiological and is designed to promote survival. Chronic or unpredictable stressors are deleterious and contribute to several neurodegenerative and psychiatric diseases. In contrast, short predictable stressors can be beneficial for cognition and emotion. Clinical and experimental studies have shown that deleterious stress negatively impacts the immune function in adults (Dhabhar, 2014; Seiler et al., 2020). Stress has been linked to the development of inflammatory diseases such as inflammatory bowel disorder (Brzozowski et al., 2016; Sun et al., 2019), and neurons can release inflammatory molecules in response to stress and induce neuroinflammation (Black, 2002). It is also not surprising that stress has been linked, and may trigger, several psychiatric disorders such as posttraumatic stress disorder, anxiety disorders, depression and schizophrenia, and also the evolution of neurodegenerative diseases (McLeod et al., 2001; Bottaccioli et al., 2019).

Moreover, the fetal basis of adult disease hypothesis introduced the concept that adult health and behavior are programmed *in utero* and shaped throughout life by exposure to new and previously experienced stressors *via* epigenetic mechanisms (Barker et al., 1993; Calkins and Devaskar, 2011; Argyraki et al., 2019). Epigenetic mechanisms are changes in DNA methylation and histone modification, which do not modify the genetic code but modulate transcription and translation, reinforcing or inhibiting some genes, and regulate when and where corresponding proteins are expressed (Lejarraga, 2019). These genes alter programming, thereby modifying responses following stimulation of metabolic and hormone regulators. Changes in gene expression may affect metabolic responses throughout life. For example, prenatal exposure may contribute to health problems that arise later in life such as obesity, diabetes, cardiovascular disease, cancer, and PD. Epigenetic changes persist even when the original triggering conditions are no longer present (Ho and Tang, 2007; Lejarraga, 2019). In line with this, juvenile and early life stresses have been linked to long term meta-plasticity-like effects on inflammatory responses in adulthood, and this memory may increase susceptibility to neurodegenerative diseases in adult life (Shtoots et al., 2018). This enhanced inflammatory response to stressors is called behavioral meta-plasticity. These findings mean that early life stress can produce long lasting changes in the immune response and increased pro-inflammatory cytokine and chemokine expression, and increased recruitment of innate immune cells (Carpenter et al., 2010; Lopes et al., 2012). Furthermore, stress *in utero* can increase the susceptibility for excessive neuroinflammation, anxiety and neurodegeneration in adulthood (Desplats et al., 2019), which has been observed in both animal models and humans (Jawahar et al., 2015; Van den Bergh et al., 2017; Frasch et al., 2018). Finally, the association between childhood trauma and plasma inflammatory biomarkers have been observed among 1,037 members of the Dunedin Multidisciplinary Health and Development Study through a longitudinal prospective study (Danese et al., 2007). Study members have been followed for 45 years since they were born in 1972–1973 in Dunedin, New Zealand (Poulton et al., 2015). This study showed that cumulative experience to childhood maltreatment was associated with significantly elevated inflammation in adult life, with increased CRP and fibrinogen levels and leukocyte count (Danese et al., 2007). Other studies have subsequently tested this association, which is confirmed by meta-analysis reviews (Coelho et al., 2014; Baumeister et al., 2016). In this context, it would be advantageous to reduce or eliminate deleterious stress from our lives and raise public awareness of the effect early life stress, maltreatment, and bullying has on mental health and wellbeing throughout the lifespan.

An association between loneliness and inflammation has been found and is currently of great interest due to the impact of the COVID-19 pandemic on social isolation. In animal models, chronic stress followed by social isolation promotes depression by increasing microglia and astrocyte activity and reduced hippocampal neurogenesis in mice (Du Preez et al., 2021). More clinical and participatory action research needs to examine the impact of social isolation on neuroinflammation and the development of psychiatric and neurodegenerative diseases. Epidemiological studies obtained during or in the first 2 years after the COVID-19 pandemic will add information to this field.

Mental health and wellbeing can be improved by individuals being aware of, and responding effectively, during periods of high stress. Increasingly, evidence supports the use of mindfulness meditation, and yoga, which are commonly used to manage wellbeing, and these practices decrease inflammation (Twal et al., 2016; Pascoe et al., 2017). For example, a recent meta-analysis study mentioned that breathing, meditation, yoga, and Tai Chi practice downregulated pro-inflammatory genes and NF-κB pathway (Buric et al., 2017).

Mindfulness is a way of paying attention to the present state, which originated in the eastern meditation practices in Buddhism and has been described as “paying attention in a particular way, on purpose, in the present moment and non-judgmentally” (Kabat-Zinn, 2009). Mindfulness meditation is an intrinsic capacity of the human mind and has only recently been highlighted because it can improve human health and wellbeing. Meditation seems to reduce blood cortisol, CRP, TNF-α, IL-6, and the transcription factor NF-κB activity across all meditation practices, acutely (measured 5–20 min after a 20 min meditation/yoga session) and chronically (3 or 4 months post-practice) (Kiecolt-Glaser et al., 2014; Creswell et al., 2016; Twal et al., 2016). A review of randomized control trials examining the effect of mindfulness on the immune system observed reduced transcription of NF-κB and CRP levels and increases in cell-mediated defenses (CD4^+^ T cell count and activity) and telomerase activity (Black and Slavich, 2016). The increased telomerase activity protects the ends of chromosomes from DNA damage and plays a central protective role in cell fate and aging, therefore, is linked to a longer and healthier life. However, this study needs to be replicated to understand the mechanisms linking mindfulness meditation and its positive effects on immunity and disease prevention. Finally, the stress-induced increase in neuroinflammation is reduced during and after mindfulness meditation (Pascoe et al., 2017).

Several recent studies provide evidence that yoga reduces the harmful effects of stress and inflammaging. Yoga for 12 weeks slowed cellular aging and increased anti-inflammatory cytokines, and diminished pro-inflammatory cytokines and cortisol levels (Tolahunase et al., 2017), with participants feeling less depressed and anxious (Cahn et al., 2017). A systematic review of randomized controlled trials mentioned that yoga decreases pro-inflammatory cytokines IL-1β, TNF-α, and IL-6 and should be part a complementary intervention for people at risk of diseases with an immunological component (Falkenberg et al., 2018). The length of individual sessions varied from 30 to 90 min; daily–once per week; and yoga was practiced for 1–6 months, with most studies including a 8–12 week yoga program. Ideally, yoga should be practised regularly throughout life to produce a consistent decrease in pro-inflammatory cytokines (Lurie, 2018; Magan and Yadav, 2020). These studies show that yoga may reduce neuroinflammation and the risk of neurodegenerative and psychiatric disorders in people living with chronic stress.

### 4.5. Quality of the air we breathe and spending time in natural environments

Most people live in an urban society with artificial environments containing moderate levels of pollution. Pollution is composed of particulate matter, ozone (O_3_), carbon monoxide (CO), sulfur dioxide (SO_2_), nitrogen oxide (NO), and lead (Pb). These pollutants are stated to be hazardous to human health by the Environmental Protection Agency and are the most prevalent environmental risk factors linked to increased inflammation (Craig et al., 2008). These pollutants have also been linked to increased neuroinflammation and related neurodegenerative diseases such as PD and AD in humans and animal models (Levesque et al., 2011a,b; Jankowska-Kieltyka et al., 2021). Analysis of brain tissues from people living in highly polluted areas shows increased levels of pro-inflammatory markers such as IL-1β and COX2, and BBB damage (Calderon-Garciduenas et al., 2008). Microglia are also chronically activated by either pro-inflammatory stimuli or in response to neuronal damage. There are three mechanisms underlying these effects (Block and Calderón-Garcidueñas, 2009). First, components of air pollution may directly activate microglia. Second, pro-inflammatory cytokines from the peripheral systemic inflammatory response can trigger neuroinflammation. Third, particles or cytokines derived from the periphery may damage neurons, which in turn activate microglia. For example, a study showed that chronic exposure to particulate matter in Mexico City increased oxidative stress, neuroinflammation, and innate and adaptive immune responses in children’s brains leading to pathologies similar to those observed in PD and AD (Fonken et al., 2011). In support of this, inhalation exposure to air pollutants found in traffic triggers increased activity of matrix metalloproteinases (MMP) and degradation of tight junction proteins in mouse brain vasculature resulting in increased BBB permeability and an increase in neuroinflammation (Oppenheim et al., 2013).

Conversely, populations with higher greenspace exposure are more likely to have good overall health (Lee and Maheswaran, 2011; White et al., 2019). Studies show that walking or exercise in nature improves cognition and mood in people with major depressive disorder (Berman et al., 2012) and also increased self-esteem (Barton and Pretty, 2010). Moreover, being in nature reduces stress, decreases exposure to pollution and increases sleep duration–factors known to reduce neuroinflammation (see Sections “4.4. Mental health and wellbeing,” “4.5. Quality of the air we breathe and spending time in natural environments,” and “4.7. Importance of quality sleep”). Studies in countries with dense populations show that spending time in natural environments such as a forest, reduced levels of cortisol, increased levels of protective immune function (levels of NK cell activity) and reduced pro-inflammatory cytokines such as IL-6 and TNF-α (Miyazaki et al., 2011; Mao et al., 2012) by decreasing stress responses (e.g., reducing heart rate and blood pressure) and favoring a relaxed state. Spending time outside, in forests, parks, mountains and oceans should be part of a healthy lifestyle and is sometimes recommended by doctors. In fact, in Scotland, medical doctors are now prescribing time spent in natural environments as treatments (Carrell, 2018; Koselka et al., 2019). However, green spaces need to be accessible for everyone. Therefore, city designs need to include safe green spaces so that all residents can maintain positive health.

### 4.6. Exposure to pesticides

Pesticides are widely used in the agricultural industry as well as in houses and offices to control weeds and insect manifestations. Serious health concerns have been raised about occupational exposure to pesticides and from residues found in/on food and drinking water. Pesticides last a long time and are degraded slowly. Occupational exposure in agricultural, pesticide and extermination industries can be high, whereas home exposure through eating food and drinking water is variable. Harmful effects depend on the toxicity of the pesticide, preventative measures taken during its application, dose, and persistence of pesticide residues in the environment and ongoing exposure (Damalas and Eleftherohorinos, 2011).

Accumulating experimental and epidemiological evidence shows that the pathogenesis of many chronic neurodegenerative (PD, AD, MS, HD, and ALS) and psychiatric (depression, anxiety, cognitive impairment, and autism) disorders are exacerbated by pesticide exposure (Dhaini, 2009; Parrón et al., 2011; Vinceti et al., 2017; Arab and Mostafalou, 2022). Exposure to pesticides can trigger damaging immune system effects and induce neurotoxicity (Costa et al., 2013; Chen et al., 2014; Fenga et al., 2014; Mokarizadeh et al., 2015). Much research has been conducted on this topic and here we discuss a few examples. For a more detailed explanation of the neurotoxic effects of pesticides, epidemiological studies and neurotoxicity mechanisms, see recent reviews (Xiao et al., 2021; Arab and Mostafalou, 2022; Vellingiri et al., 2022).

Epidemiological cohort and case-control studies have correlated an increased risk for PD to develop with pesticide exposure among greenspace workers, farmers, and horticulturists (Hertzman et al., 1994; Tüchsen and Astrup Jensen, 2000; Kenborg et al., 2012). Many epidemiological studies have also linked pesticide exposure to increased risk of developing AD (Gauthier et al., 2001; Tyas et al., 2001; Hayden et al., 2010; Parrón et al., 2011; Agarwal et al., 2020; Li et al., 2021) by inducing oxidative stress, neuronal damage and neurobehavioral alterations. Pesticide exposure also increases the risk of developing MS (Parrón et al., 2011; Graves et al., 2017), ALS (Morahan and Pamphlett, 2006; Andrew et al., 2017; Povedano et al., 2018) and autism when exposure occurs during the prenatal period (Brown et al., 2018).

Inflammation is a common mechanism of pesticide-induced neurotoxicity. In rodents, exposure to the pesticides paraquat and rotenone cause behavioral impairments, increase ROS and the pro-inflammatory cytokine TNF-α in the substantia nigra, and these neuroinflammation changes lead to the degeneration of the nigrostriatal dopaminergic system and parkinsonian motor symptoms (Sherer et al., 2003; Hutson et al., 2011; Mitra et al., 2011). Therefore, paraquat and rotenone have been used to model PD in many animal studies (Uversky, 2004; Nisticò et al., 2011). Similarly in animal models of AD, paraquat, chlorpyrifos, and dichlorodiphenyldichloroethylene increase production of ROS, causing neuronal death and degeneration (Tang, 2020). In a striatal neuron model of HD, the pesticide chlorpyrifos induces oxidative stress by production of ROS and neurotoxicity (Dominah et al., 2017). ROS induced by pesticides can trigger NLRP3 inflammasome in microglia leading to the production of IL-1β, exacerbating neuronal death and function (Moloudizargari et al., 2019), which is one mechanism linking pesticides and neuroinflammation. Rotenone induces transcriptional changes *in vitro* similar to those observed in patients with autism such as free radical production and disrupts microtubules in neurons (Pearson et al., 2016). Moreover, rotenone activates microglia and astrocytes and increases pro-inflammatory cytokine production, which attach to cytokine receptors and initiate neurotoxic intracellular mechanisms and generation of iNOS and oxidative stress, damaging neurons. Finally, pesticides have also been shown to increase gut inflammation and alter the composition of the gut microbiome, which induces neuroinflammation through the gut-brain axis (see Section “4.2. The gut-brain axis”) and is linked to neurological diseases (Chiu et al., 2020).

Experimental and epidemiological evidence shows a link between pesticide exposure and the increased incidence of developing neurodegenerative and psychiatric diseases, with neuroinflammation being an important common mechanism inducing neurotoxicity. Agriculturalists and health experts understand the risks of pesticide exposure and many people are advocating for greater controls and reduced use of pesticides. This is an important first step. However, because health effects for most people are cumulative over a lifetime, it will take several decades before a population-based reduced risk of neurodegenerative and psychiatric disorders will be evident.

### 4.7. Importance of quality sleep

People living with neurodegenerative diseases, such as AD, PD, and MS, as well as psychiatric disorders such as depression and anxiety, experience sleep disorders. These conditions can induce a sleep disorder, but the genesis may also be that a sleep disorder contributes to brain disorders. Previous studies show that poor sleep simulates peripheral immunity by increasing circulating pro-inflammatory cytokine levels, increasing inflammatory signaling pathways and increasing innate immunity (Marshall and Born, 2002; Opp and Krueger, 2015; Qazi and Farraye, 2018). Sleep loss and disrupted sleep have been shown to induce acute (Irwin et al., 2010) and chronic inflammation (Hurtado-Alvarado et al., 2016). For example, insufficient sleep quantity facilitates and/or exacerbates pain in healthy volunteers through elevation of circulating IL-6 (Haack et al., 2007). Interestingly, 64 h of sleep deprivation increased leukocyte and natural killer cell function, which was reversed by sleep, suggesting that sleeping has the potential to reverse adverse effects of inflammation (Dinges et al., 1994). Recently, sleep disorders have been considered a causal part of neuroinflammation found in neurodegenerative and psychiatric disorders. Reduced sleep has been associated with increased secretion of pro-inflammatory cytokines such as TNF-α in the blood (Vgontzas et al., 2004; Irwin, 2015), with microglia activation playing a key role (Wisor et al., 2011; Nadjar et al., 2017). Short term (6 h) sleep deprivation causes a significant increase in B cells in the brain and elevates expression of the migration-related C-X-C chemokine receptor type 5 (CXCR5) on B cells and its ligand CXCL13 in the meninges in mouse brains (Korin et al., 2020). B cells have cytokine-producing states and are antigen presenting cells, in addition to their antibody production function (Tarlinton, 2019). Therefore, the neuroinflammation that occurs due to reduced sleep quality with neurodegenerative and psychiatric disorders exacerbates these conditions (Ahnaou and Drinkenburg, 2021).

### 4.8. Cannabinoids

Preparations of the cannabis plant *Cannabis sativa* have been used for thousands of years by different cultures for many purposes, including medicinal properties. Its sedative and psychotropic effects mean the plant is used as a recreational drug throughout the world. Cannabinoids have been studied for their therapeutic properties in neuroinflammatory diseases. However, legal systems have categorized drugs as being socially acceptable (alcohol and nicotine) or unacceptable (cannabis and others), which has slowed research examining the anti-inflammatory properties of cannabinoids. Cannabidiol, the main non-psychotropic component, exerts anti-inflammatory effects by inhibiting the synthesis and release of pro-inflammatory molecules, like cytokines, NO and glial fibrillary acidic protein from activated astroglia (Esposito et al., 2007) through the peroxisome proliferator-activated receptor gamma (PPARγ) (Esposito et al., 2011). This is associated with inhibition of p38 mitogen-activated protein kinase (MAPK) and regulation of NF-κB, which controls transcription of pro-inflammatory factors (Esposito et al., 2006). Moreover, controlling these pro-inflammatory molecules regulates microglia migration, which is involved in neuroinflammation, preventing recruitment of microglia to lesion sites (Walter et al., 2003). Cannabinoids act directly on cannabinoid type 1 and type 2 (CB1 and CB2) receptors, transient receptor potential cation channel subfamily V member 1 (TRPV1) largely distributed within the CNS. A recent review shows that cannabinoid binding to CB1, CB2, and TRPV1 is neuroprotective, decreases TNF-α (CB1 and 2) and IL-12 (CB1), inhibits chemokine production by astrocytes (CB2), and reduces proliferation (CB2), migration (CB2) and activates (TRVP1) microglia (Antonazzo et al., 2019). These findings are promising as an agent for delaying the progression of neurodegenerative diseases such as HD and PD. However, clinical trials are needed to examine the efficacy of cannabinoids to treat neurodegenerative and psychiatric disorders.

### 4.9. Effect of tobacco smoking

Tobacco smoking is a worldwide epidemic, a significant cause of death and morbidity (Sopori, 2002), and directly induces cardiovascular diseases, lung cancer and chronic obstructive pulmonary disease. In the context of (neuro)inflammation, smoking’s effects are complex and can be protective, as well as detrimental, depending on the disease. In MS, smoking enhances inflammatory responses resulting in an increased risk of developing the disease (Alrouji et al., 2019). Smoking is also a risk factor for dementia and past exposure induces neuroinflammation and aggravates cognitive impairment *via* NLRP3 and eukaryotic translation initiation factor 2A pathways in animal models (Meng et al., 2020). However, tobacco smokers have a reduced incidence or delayed onset of PD (Thacker et al., 2007; Ritz et al., 2014), and acute smoking suppresses inflammatory cytokines and has also been considered protective for neuroinflammation in PD. Smoke contains numerous chemicals that could be responsible for protective effects. Unless the exact protective product from tobacco is removed from its constituents and administered in a safer way, tobacco smoking should be avoided because it increases the risks for other diseases.

### 4.10. Exposure to metals

Metals, such as iron (Fe), copper (Cu), manganese (Mn) and chromium (Cr) are essential for normal cell metabolism when kept at homeostatic levels. To achieve this, complex mechanisms regulate intracellular and extracellular concentrations of these metals. When this process is dysregulated, it is called dyshomeostasis of essential metals, and this leads to increased oxidative stress, production of ROS, activates microglia and overproduction of the pro-inflammatory cytokines IL-1β and TNF-α, leading to neuroinflammation. On the other hand, the presence of non-essential heavy metals such as lead (Pb), aluminum (Al), cadmium (Cd), and mercury (Hg) have direct neurotoxic effects on the brain and are not readily detoxified by immune mechanisms, activate glia and increase production of pro-inflammatory cytokines leading to a chronic inflammatory state. For example, including aluminum in drinking water promotes neuroinflammation in experimental models (Campbell et al., 2004; Becaria et al., 2006).

Both essential and non-essential metals induce neuroinflammation, contributing to neurodegenerative and psychiatric disorder pathology (Mason et al., 2014). For both PD and AD, dysregulated metal brain homeostasis (Fe, Cu, and Zn) may be part of the disease pathogenesis through neuroinflammation (Perry et al., 2003; Das et al., 2021; Nakagawa and Yamada, 2022). In MS, excessive neuroinflammation may increase Fe deposition (Williams et al., 2012). In AD, both Pb and Hg induce glia reactivity and neuroinflammation, and have been linked to the disease (Monnet-Tschudi et al., 2006; Siblerud et al., 2019), and extracellular amyloid-beta plaques contain excessive essential metals Cu, Fe, and Zn and induce neuroinflammation. Several epidemiological studies have linked chronic metal exposure (Hg, Pb, Mn, Cu, Fe, Al, bismuth, titanium, and Zn) to the risk of developing PD (Gorell et al., 1997, 1999; Miller et al., 2003; Lucchini et al., 2007; Raj et al., 2021).

Avoiding heavy metal exposure is a prevention strategy that reduces the neuroinflammation often underlying neurodegenerative and psychiatric disorders. When prevention is not feasible, metal chelators could be used to minimize diseases outcomes.

## 5. Conclusion

Neuroinflammation is an important parameter underlying neurodegenerative and psychiatric disorders, therefore, management is critical for developing strategies to treat them. Drugs targeting neuroinflammation are on the market, but other drugs, lifestyle changes and natural anti-inflammatory compounds produce promising results. A comprehensive, recent review has summarized neuroinflammatory treatment strategies for PD (Kip and Parr-Brownlie, 2022). Effective prevention or delayed onset of neurodegenerative or psychiatric disorders requires having biomarkers to know when and where in the CNS neuroinflammation is occurring. Furthermore, to prevent side effects, drugs may need to be targeted to particular brain regions and cell types. Such therapies are likely decades away.

Some interventions are available now if specific biomarkers are available. Therefore, these strategies could be implemented early to prevent or delay the onset of disease. Interventions may need to be applied during specific periods (e.g., prenatally), throughout the lifespan and/or when neurodegenerative or psychiatric diseases are likely to appear, e.g., late teens for schizophrenia or mid-life for PD and AD. Given that there are no drugs that currently halt or reverse most neurodegenerative diseases, prevention and/or acting early is a valid strategy. Balance is key. Some solutions need to be solved at the population level, i.e., reducing pollution and climate change; population-based strategies to modify such factors could potentially result in fewer cases of inflammatory-related diseases. At system levels, recategorizing drugs by medicinal effects could enable greater modulation of neuroinflammation and inflammation-related diseases. Other solutions are primarily under individual control. Dietary strategies, ongoing moderate physical activity, moderate alcohol consumption, reducing stress and spending time outdoors in natural environments, adopting meditative/mindfulness strategies, and having enough quality sleep should be encouraged. Here, effective public health policies may have a role at the population level, e.g., removing taxes on fresh fruits and vegetables and quality protein sources so they are affordable. While this review focused on neurodegenerative and psychiatric disorders, neuroinflammation is present in neurological disorders and similar strategies could also produce positively outcomes.

For many individuals, translating knowledge into behavioral changes and sustaining that for life is a barrier, constituting a large research field and many commercial products designed to help the process. However, maintaining a healthy lifestyle in a busy Western work environment is difficult due to accumulating effects of stress, anxiety, depression and chronic lack of sleep. Physical and mental wellbeing is pivotal. In this context, wellbeing should be a focus of daily life and prioritized by doctors, employers, politicians, and people in positions of influence. One strategy that could be adopted is a reduced working week, which increases work productivity overall and restores a better work-life balance (Ivancevich, 1974; Foster et al., 1979; Haar et al., 2014; Kossek et al., 2014; Kamerâde et al., 2019; Laker and Roulet, 2019). However, employers are slow to adopt this strategy.

Lifestyle factors can be harnessed to reduce the population risk of neurodegenerative and psychiatric disorders by modulating neuroinflammation. As more research is produced, the benefits of lifestyle interventions may be accurately quantified for some disorders. Finally, for this knowledge to have impact, be adopted and translated to refine treatment regimes, the mechanisms need to be shared with clinicians and people living with these disorders.

## Author contributions

All authors listed have made a substantial, direct, and intellectual contribution to the work, and approved it for publication.
